# Identification of Representative Equivalent Volumes on the Microstructure of 3D-Printed Fiber-Reinforced Thermoplastics Based on Statistical Characterization

**DOI:** 10.3390/polym14050972

**Published:** 2022-02-28

**Authors:** Thiago Assis Dutra, Rafael Thiago Luiz Ferreira, Hugo Borelli Resende, Luís Miguel Oliveira, Brina Jane Blinzler, Leif E. Asp

**Affiliations:** 1 DPS—Product Systems Development, INEGI—Institute of Science and Innovation in Mechanical and Industrial Engineering, 4200-465 Porto, Portugal; loliveira@inegi.up.pt; 2 GPMA—Research Group on Additive Manufacturing, DCTA ITA IEM, ITA—Aeronautics Institute of Technology, São José dos Campos, São Paulo 12228-900, Brazil; rthiago@ita.br (R.T.L.F.); hbrinnovation@gmail.com (H.B.R.); 3 Arris Composites, Berkeley, CA 94710, USA; brina.blinzler@gmail.com; 4 Division of Material and Computational Mechanics, Department of Industrial and Materials Science, Chalmers University of Technology, SE-412 96 Gothenburg, Sweden; leif.asp@chalmers.se

**Keywords:** polymer–matrix composites (PMCs), mechanical properties, computational mechanics, 3D printing, representative volume element (RVE)

## Abstract

The present work describes a methodology to compute equivalent volumes representing the microstructure of 3D-printed continuous fiber-reinforced thermoplastics, based on a statistical characterization of the fiber distribution. In contrast to recent work, the methodology herein presented determines the statistically equivalent fiber distribution directly from cross-section micrographs, instead of generating random fiber arrangements. For this purpose, several regions, with different sizes and from different locations, are cropped from main cross-section micrographs and different spatial descriptor functions are adopted to characterize the microstructures in terms of agglomeration and periodicity of the fibers. Detailed information about the adopted spatial descriptors and the algorithm implemented to identify the fiber distribution, as well as to define the location of cropped regions, are given. From the obtained statistical characterization results, the minimum size of the equivalent volume required to be representative of the fiber distribution, which is found in the cross-section micrographs of 3D-printed composite materials, is presented. To support the findings, as well as to demonstrate the effectiveness of the proposed methodology, the homogenized properties are also computed using representative equivalent volumes obtained in the statistical characterization and the results are compared to those experimentally measured, which are available in the literature.

## 1. Introduction

Over recent years, the Additive Manufacturing (AM) of reinforced polymers has been playing an important role in the production of high-performance components, opening the door for new applications in the manufacturing of lightweight structures. Inserted into the material extrusion-based category, the Fused Filament Fabrication (FFF) [1,2,3,4,5,6], often referred to by the term 3D printing, is an Additive Manufacturing process that can work with continuous fiber-reinforced thermoplastics [7,8,9,10,11,12,13,14,15,16,17,18,19,20,21]. From a mechanical behavior point of view, both the printing process parameters and individual constituent characteristics, e.g., fiber distribution, affect the resulting performance of 3D-printed composite materials [22,23,24,25,26]. Since experiments for determining the mechanical properties of composite materials are relatively expensive, several approaches have been employed, either for predicting the resulting mechanical properties or guiding the setup of experiments. In regards to the available techniques for predicting the mechanical properties of 3D-printed composite materials, recent work has reported the use of classical mean-field approaches although only longitudinal elastic properties (along fiber direction) were computed and compared with experimental results [7,8,9,10,11]. On the other hand, different numerical techniques can be found in the available literature to predict the mechanical properties of traditional composite materials. In this context, computational homogenization techniques are very suitable to describe the mechanical behavior of heterogeneous materials, mostly when this behavior is difficult to be obtained experimentally. Generally, a sub-volume which is representative of a whole is discretized using finite element methods, consequently reducing the simulation time with good results [27,28,29,30,31,32,33,34,35]. On the other hand, fractal solutions are also able to determine different characteristics of fibrous porous media [36,37].

To improve the prediction of the mechanical behavior of heterogeneous materials, recent studies have presented methodologies to create sub-volumes with random microstructures which attempt to capture the effects of fiber arrangement on the homogenized properties, and also on the distribution of the stresses at microscopic level [38,39,40,41,42,43,44,45,46,47,48]. These approaches are very interesting from the point of view of more realistic representations when compared to classical periodic fiber arrangement. However, they assume that the microstructure of a composite material is completely random regardless of other factors, e.g., the manufacturing process which can affect the fiber spatial and pores distribution [49,50]. Furthermore, this strategy may not provide suitable results when fiber-reinforced materials with relatively low fiber volume fractions, ca. 32%, are analyzed, which is the case of the continuous fiber-reinforced 3D-printed materials [7,13]. In general, traditional pre-impregnated composite materials present a higher fiber volume fraction which results in fiber arrangements better distributed than those seen in 3D-printed composite materials.

### Objective and Contributions

It is verified from the literature that a different strategy should be applied to define an equivalent microstructure of 3D-printed fiber-reinforced composite materials, mostly when they present relatively low fiber volume fractions. In this context, it is believed that a methodology to obtain equivalent microstructures directly from cross-section micrographs, which are determined according to spatial descriptors functions, is more suitable for this purpose. Additionally, it is also believed that adopting this strategy some particularities inherent to the manufacturing process could be contemplated in the models. Based on this line of thought, the authors introduced a methodology in a previous work [26] and information was given about the size of the equivalent microstructures, as well as the adopted spatial descriptor functions. However, important information about the complete image processing methodology, as well as specific results of the fiber distribution characterization, could not be detailed. In view of these aspects, the present work aims to describe in detail the methodology applied to obtain statistically equivalent volumes representing the microstructures found in 3D-printed continuous fiber-reinforced composites. To demonstrate the effectiveness of the methodology, the homogenized elastic properties are computed using the equivalent microstructures computed using the proposed algorithm. The numerical results are then compared to those experimentally measured that are available in the literature.

Among the advantages of employing the proposed methodology, the capability to include characteristics inherent to the manufacturing process, the low computational cost, and the flexibility to identify different types of microstructures can be highlighted. In addition, the methodology herein presented is highly suitable for parametric studies related to several aspects involving micromechanics. For instance, given a set of cross-section micrographs, the proposed methodology could provide parameterized equivalent microstructures to determine the stresses and strains at microscopic level as well as to study the mechanisms of failure acting at the constituent level. In addition, a parameterized study involving the interface between fiber and matrix could also be possible. In this case, the interface size could be parameterized in function of the size of the fibers found by the algorithm, and the influence of its main characteristics could be evaluated.

Regarding paper structure, detailed information about the adopted spatial descriptors, as well as about the process of fiber distribution identification and cropped regions definition, i.e., the proposed algorithm for image processing, are given in Section 2 and Section 3. The application of the proposed algorithm is presented in Section 4 according to parameters defined in function of the fiber diameter. In this parametric analysis, a bank of images is generated from three cross-section micrographs previously obtained. The results obtained from the statistical characterization are detailed in Section 5, where the minimum size of a representative microstructure for 3D-printed continuous fiber-reinforced composite materials is given in terms of the parameters previously defined. Lastly, the homogenized properties are predicted using the obtained equivalent microstructures and the results are presented in Section 6, where the effect of the equivalent fiber distribution on the homogenized elastic properties is also discussed. To support the discussion, the obtained numerical results are compared to those experimentally measured.

## 2. Adopted Spatial Descriptor Functions

Recent investigations [39,41,42,47,51,52,53,54,55] applied different statistical techniques for characterizing spatial distributions of individuals in populations. These individuals, frequently represented by points in a certain arrangement, are viewed in the context of fiber-reinforced composite materials as the center of fibers distributed in the cross-sectional area of unidirectional composites. Among the techniques, the computation of Voronoi polygon areas and neighboring distances, as well as the calculation of the nearest neighbor distances and the second-order intensity function have been widely applied on the composite materials field [39,51,52,55].

In general, these statistical techniques can provide information about the periodicity and/or the agglomeration of individuals in a distribution, which are then used to quantitatively characterize them. For instance, Ref. [39] noted that the standard deviation of the areas represented by the Voronoi polygons gives information about the periodicity of the distribution, i.e., in a periodic distribution the standard deviation is zero since all polygons are equal and consequently have the same area. The neighboring fiber distances, which is determined by the average distance between an individual and its neighbors, is also a measure of the periodicity of a distribution. Analogously to the characterization based on the Voronoi polygons, in a periodic distribution the standard deviation of the computed neighboring distances is also zero.

From the literature [39,51,52], it can be seen that the probability density function of the smallest distance from one individual to its neighbors is normally computed in the nearest neighbor distance technique. In addition, the second and third nearest neighbor distances may also be computed providing more information about the interaction between the individuals. For its part, the second-order intensity function was considered by some authors [39,56] as one of the most informative descriptors of spatial distributions providing information about the periodicity and agglomeration.

Since all techniques presented above have been demonstrated to be effective in the quantitative characterization of spatial distributions, the present work focuses on the nearest neighbor distance and the second-order intensity function to quantitatively characterize the fiber arrangement obtained from cross-section micrographs of 3D-printed carbon fiber-reinforced thermoplastic composites. It is worth noting that the approach assumed herein for characterizing the fiber arrangement in cross-section micrographs using the nearest neighbor distance, is particularly different from that generally adopted in the literature. Detailed information is given as follows.

### 2.1. Nearest Neighbor Distance

As mentioned before, the neighboring fiber distances can be determined by the average Euclidean distance between an individual and its neighbors. Thus, let $P=\left\{p_{1}\left(x_{1},y_{1}\right),p_{2}\left(x_{2},y_{2}\right),\cdots ,p_{n_{p}}\left(x_{n_{p}},y_{n_{p}}\right)\right\}$ be a set of $n_{p}$ points containing the centers of fibers, distributed in the cross-sectional area of a unidirectional fiber-reinforced composite. Thus, the nearest neighbor distance ${\hat{d}}_{i}$ between a fiber $p_{i}\in P$, with center $\left(x_{i},y_{i}\right)$, and its neighboring fibers $p_{j}\in P$, with centers $\left(x_{j},y_{j}\right)$, can be written as [26]

(1) $${\hat{d}}_{i}\left(p_{i},p_{j}\right)=\min_{p_{i},p_{j}\in P}\left\{\sqrt{{\left(x_{i}-x_{j}\right)}^{2}+{\left(y_{i}-y_{j}\right)}^{2}}\right\}\;\forall i\neq j,$$

where the indices *i* and *j* are defined as $i,j=1\cdots n_{p}$.

The probability density function of the nearest neighbor distances $\hat{d}$ obtained for all points p∈P is typically computed to provide information about the periodicity of a certain distribution. For instance, the probability density function plot of the nearest neighbor distances computed for a set of points in a periodic arrangement, e.g., square or hexagonal, would be a straight vertical line since all the nearest neighbor distances are equal. On the other hand, in a random distribution the probability density function is expected to follow a Gaussian distribution [55]. According to Melro et al. in [39], information about clustering in a distribution can be also obtained verifying if the probability density function plot of the nearest neighbor distances exhibits a peak for a specific distance followed by a steep decrease.

Following the methodology previously introduced in [26], instead of computing the probability density function, the mean and the standard deviation values of the nearest neighbor distances, necessary to compute the probability density function, are analyzed separately. Defining $r_{f}$ as the radius of fibers in a distribution, the mean value $\hat{\mu }\left(\hat{d}/r_{f}\right)$ tends to 2 if the fibers are in a condition of maximum agglomeration. Higher values of $\hat{\mu }\left(\hat{d}/r_{f}\right)$ indicate that the fibers in the distribution are more spaced or dispersed. Although the ratio $\hat{\mu }\left(\hat{d}/r_{f}\right)$ of the nearest neighbor distances for an extreme case of maximum agglomeration also gives information about periodicity, the standard deviation of the nearest neighbor distances $\hat{\sigma }\left(\hat{d}\right)$ is computed for this purpose. If $\hat{\sigma }\left(\hat{d}\right)$ tends to 0 it can be verified that the fibers are arranged in a periodic distribution, since all the nearest neighbor distances are equal. The more distant from zero, the less periodic is the distribution. Table 1 summarizes the criteria adopted for quantitatively characterizing a fiber distribution using the nearest neighbor distance.

Since the spatial descriptor functions are applied in the present work to determine fiber arrangements equivalent to the fiber distributions found in the cross-section micrographs of 3D-printed composite materials, the criteria herein adopted is shown to be more convenient, given that the agglomeration and periodicity of the fibers are computed separately and the computation of second and third nearest neighbor distances are not required. Moreover, it is possible to compute the mean and the standard deviation values of the nearest neighbor distances for several small portions, i.e., small cropped regions, of a cross-section micrograph and compare the results with those obtained for the whole cross-section micrograph. In this case, the minimum size of a cropped region would be that whose values are converged to those obtained for the whole cross-section micrograph.

### 2.2. Second-Order Intensity Function

The second-order intensity function, also referred to in the literature as Ripley’s K-Function, is a well-known spatial descriptor of individuals in a population [57]. In the context of fiber-reinforced composite materials, the second-order intensity function can be defined as a ratio between the number of fibers expected to lie within a radial distance $h_{k}$ from an arbitrary fiber, and the number of fibers per unit of area.

Thus, let $P=\left\{p_{1}\left(x_{1},y_{1}\right),p_{2}\left(x_{2},y_{2}\right),\cdots ,p_{n_{p}}\left(x_{n_{p}},y_{n_{p}}\right)\right\}$ be a set of $n_{p}$ points, which represents the centers of fibers distributed in the cross-sectional area of a unidirectional fiber-reinforced composite, $L=\left\{l_{1},l_{2},\cdots ,l_{n}\right\}$ a set of line segments delimiting the region of study and *A* the area of study, the second-order intensity function $K(p,h_{k},L)$, for a given radial distance $h_{k}$ and center points p∈P, can be defined as [26,51,57]

(2) $$K\left(p,h_{k},L\right)=\frac{A}{n_{p}^{2}}\sum_{i}^{n_{p}}\sum_{j\neq i}^{n_{p}}\frac{I\left[d\left(p_{i},p_{j}\right),h_{k}\right]}{w\left(p_{i},h_{k},l_{j}\right)},$$

where the distance $d\left(p_{i},p_{j}\right)$ between the centers of fibers $p_{i}\left(x_{i},y_{i}\right)$ and $p_{j}\left(x_{j},y_{j}\right)$ is

(3) $$d\left(p_{i},p_{j}\right)=\sqrt{{\left(x_{i}-x_{j}\right)}^{2}+{\left(y_{i}-y_{j}\right)}^{2}},\;\forall j\neq i,$$

the indicator function $I\left[d\left(p_{i},p_{j}\right),h_{k}\right]$ is

(4) $$I\left[d\left(p_{i},p_{j}\right),h_{k}\right]=\left\{\begin{array}{c} 1,\;\mathrm{for}\;d\left(p_{i},p_{j}\right)\leq h_{k},\\ 0,\;\mathrm{for}\;d\left(p_{i},p_{j}\right)>h_{k}, \end{array}\right.$$

and $w\left(p_{i},h_{k},L\right)$ is a weight function that takes into account the edge effects returning the proportion of the circumference with radius $h_{k}$ contained within the region of study bounded by the line segments *L* to the whole circumference with radius $h_{k}$.

Let $d(p_{i},l_{j})$ be the distance between a point $p_{i}\left(x_{i},y_{i}\right)$ and a line segment with end points $\left(x_{1}^{l_{j}},y_{1}^{l_{j}}\right)$ and $\left(x_{2}^{l_{j}},y_{2}^{l_{j}}\right)$ given by

(5) $$d\left(p_{i},l_{j}\right)=\frac{\left|\left(y_{2}^{l_{j}}-y_{1}^{l_{j}}\right)x_{i}-\left(x_{2}^{l_{j}}-x_{1}^{l_{j}}\right)y_{i}+x_{2}^{l_{j}}y_{1}^{l_{j}}-y_{2}^{l_{j}}x_{2}^{l_{j}}\right|}{\sqrt{{\left(y_{2}^{l_{j}}-y_{1}^{l_{j}}\right)}^{2}+{\left(x_{2}^{l_{j}}-x_{1}^{l_{j}}\right)}^{2}}}.$$

Thus, the weight function $w\left(p_{i},h_{k},l_{j}\right)$ can be computed as [57]

(6) $$w\left(p_{i},h_{k},l_{j}\right)=\left\{\begin{array}{c} 1,\;\mathrm{for}\;d\left(p_{i},l_{j}\right)\geq h_{k},\\ \frac{\alpha }{2\pi },\;\mathrm{for}\;d\left(p_{i},l_{j}\right)<h_{k}, \end{array}\right.$$

where

(7) $$\alpha =2\left\{\pi -\arccos\left[\frac{d(p_{i},l_{j})}{h_{k}}\right]\right\}.$$

To simplify the notation, the second-order intensity function $K(p,h_{k},L)$ is hereinafter referred to as K(h). In a complete random distribution, the second-order intensity function $K\left(h\right)=K_{p}\left(h\right)$ is defined as [57]

(8) $$K_{p}\left(h\right)=\pi h^{2}.$$

**Figure 1:**
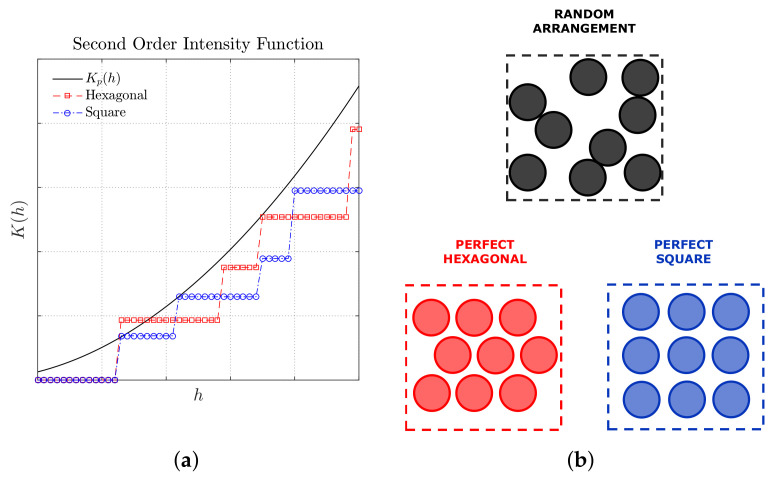
Response of the second-order intensity function K(h), or Ripley’s K-Function, expected for arrangements with same number of fibers and same area of study (**a**), distributed in random, hexagonal, and square patterns (**b**). Adapted from [52].

Taking into account the fiber distribution in the cross-sectional area of a unidirectional fiber-reinforced composite material, if the plot K(h)×h provides a monotonic positive response, the fiber distribution can be considered to be non-periodic. Moreover, comparing the plot of K(h) to the plot $K_{p}\left(h\right)$ it is possible to verify if the fibers are either dispersed or agglomerated. For instance, if $K\left(h\right)<K_{p}\left(h\right)$ it can be assumed that the distribution presents some degree of dispersion. Contrariwise, a $K\left(h\right)>K_{p}\left(h\right)$ response means that the fibers in the distribution are more agglomerated or clustered. Figure 1 shows the second-order intensity function plots expected for arrangements with same number of fibers and same area of study, distributed in random, hexagonal, and square patterns. It is possible to observe from Figure 1 the resulting stair-shaped of function K(h) for the periodic distributions in square and hexagonal arrangements. In addition, it can be realized that the fibers arranged in the periodic distributions in Figure 1 have some degree of dispersion, since their plots K(h) are under the plot $K_{p}\left(h\right)$ for different values of *h*.

In an attempt to summarize the criteria adopted for quantitatively characterizing a fiber distribution using the second-order intensity function, Table 2 describes the conditions with respect to both agglomeration and periodicity criteria.

## 3. Algorithm for Image Processing

To provide the data required for identifying representative equivalent volumes on the microstructure of 3D-printed carbon fiber-reinforced thermoplastics, the present work proposes a complete methodology to compute and analyze cropped regions, also referred to as portions, of cross-section micrographs. Additionally, the proposed methodology also provides information for building finite element models used in computational homogenization analyses. The methodology herein proposed identifies the microstructure of cropped regions supported by a fiber identification algorithm, which is based on pixels determination, and verifies if the fiber volume fractions computed from the microstructure of the cropped regions are within the defined range. In this section, the steps of the proposed methodology are described, and details are given about the generated bank of images used in the statistical characterization. It is important noting that the performance of the methodology herein proposed is directly linked to the image quality. If the image presents marks or blurred portions, the accuracy of the processed data can be compromised.

**Figure 2:**
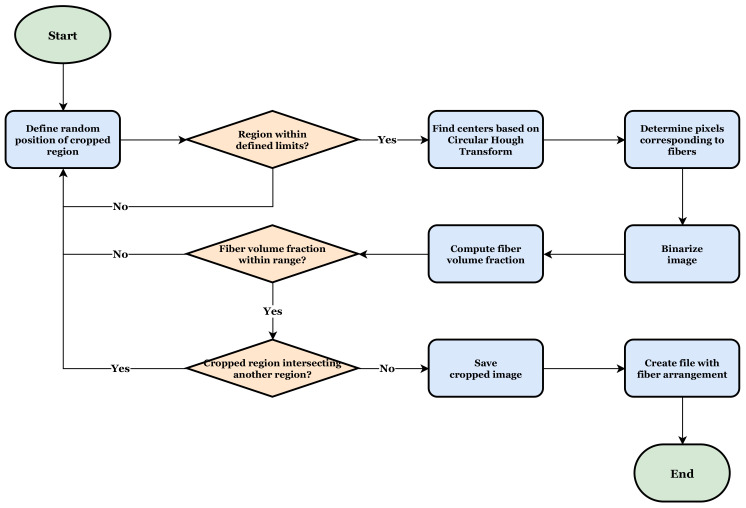
Proposed algorithm for cross-section micrograph processing.

**Figure 3:**
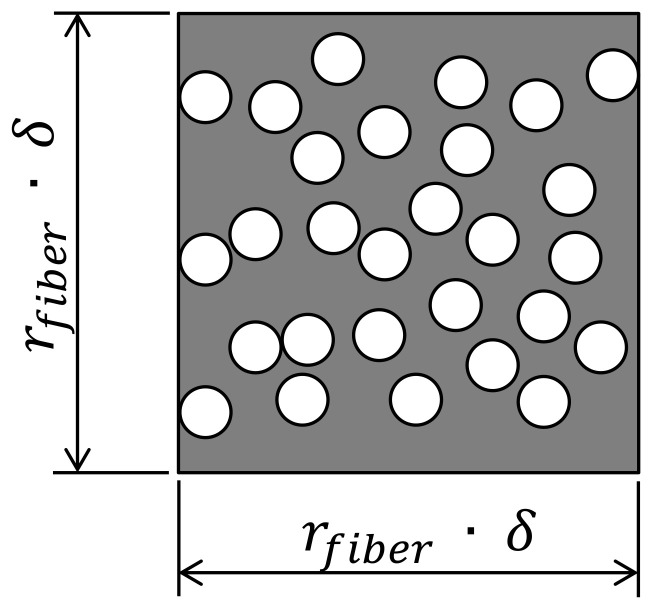
Schematic illustration of a cropped region and its size in function of the parameter δ.

The proposed methodology was implemented using commercial software MATLAB and follows the procedure presented in Figure 2. First, a cross-section micrograph of the material is read and input data about the regions to be cropped are informed. Initially, it is required to inform the mean radius $r_{f}$ of the fibers, the limits $V_{f,min}$ and $V_{f,max}$ for the targeted fiber volume fraction, the number $n_{images}$ of output cropped regions and the size δ of the cropped regions. The size δ is defined as a multiplication factor applied over the fiber radius where the edge sizes of the cropped regions are given by $l_{edge}=r_{fiber}\times \delta$. Figure 3 depicts a schematic illustration of a cropped region with edge sizes $r_{fiber}\times \delta$. A reference value for the fiber radius $r_{fiber}$ was initially measured with the support of scanning electron microscope images. In this case, a fiber radius $r_{fiber}\approx 4$ μm was found. This value was later confirmed analyzing the cross-section micrographs of the 3D-printed composite material.

Thus, in the first step of the algorithm in Figure 2 a random initial position $\left(x_{0},y_{0}\right)$ for the cropped region is defined and it is verified if the whole cropped region is within the cross-section micrograph. Otherwise, a new position is defined, and the verification is also performed. The idea behind randomly sampling the position of the cropped regions is related to the independence of the location in the microstructure that a statistic-based representative volume should have [58,59].

The second main step is related to the fiber identification. Basically, the algorithm computes the fiber distribution by identifying the material constituents, i.e., matrix and fiber, based on the Circular Hough Transform [60] and, after determining the pixels corresponding to fibers, converts the original image to a binary image, e.g., assigning 0 for the matrix and 1 for the fiber. The Circular Hough Transform was applied in the algorithm through the function *imfindcircles()* already available in MATLAB. More details about the function *imfindcircles()* is available in [61]. In addition to the inputs already defined in the previous step, the function *imfindcircles()* requires a sensitivity factor and an edge threshold. The sensitivity factor is the sensitivity for the Circular Hough Transform accumulator array. Increasing the sensitivity factor, the function *imfindcircles()* detects more circular objects, including weak and partially obscured circles. However, too high sensitivity values increase the risk of false detection. Thus, if the constituents have pixels with close values in a gray scale, the threshold shall be finely adjusted. Lower values for the edge threshold lead the function *imfindcircles()* to detect more circular objects with both weak and strong edges. When increasing the value of the threshold, it detects fewer circles. For all the cross-section micrographs analyzed in the present work, a sensitivity value equal to 0.95 and an edge threshold equal to 0.4 worked well in identifying the edges of the fibers.

In the next step, the fiber volume fraction of the cropped region is obtained computing the proportion of the number of pixels representing the fibers to the total number of pixels of cropped regions. More details about this adopted strategy are presented in following paragraphs. Depending on the position where the region is cropped from the cross-section micrograph, the fiber volume fraction may change. For instance, if the defined position lies in a portion that has a high concentration of matrix, a low value of fiber volume fraction is computed. On the other hand, if the defined position lies in a portion that has a high concentration of fiber, a high value of fiber volume fraction is then computed. Therefore, if the computed fiber volume fraction is not within the range previously defined, the algorithm goes back to the first step where a new position for the cropped region is defined. If the computed fiber volume fraction is within the defined range, it is then verified if the cropped region is intersecting, or overlapping, another cropped region previously defined. This step is particularly important to avoid the similarity between the cropped regions ensuring the diversity of images in the generated bank.

**Figure 4:**
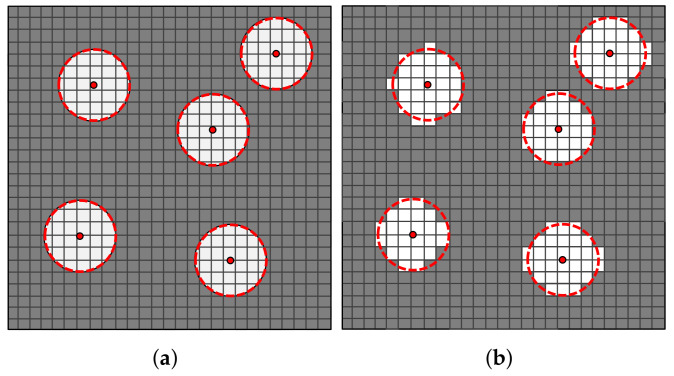
Representation of a fiber/matrix distribution with deviations between the expected area for the whole circumference (**a**) and the area of a set of pixels within a circumference (**b**). The white squares represent the pixels corresponding to the fibers and the gray squares represent the pixels corresponding to the matrix. The red dashed lines represent the circles obtained with the support of MATLAB function *imfindcircles()*.

Lastly, the cropped image is saved and an output file, which is used as an input data to computational homogenization analyses, is written. This output file contains the geometrical position and the pixels assigned values which corresponds to the material constituents, e.g., 0 for the matrix and 1 for the fibers. As mentioned before, in the proposed algorithm the fiber volume fraction is obtained computing the proportion of the number of pixels representing the fibers to the total number of pixels of cropped regions. If the resolution of the image is particularly fair or the size of the cropped region is relatively small, this strategy may provide some values for the fiber volume fraction that may not represent those experimentally measured. The deviations which may appear are depicted in Figure 4. It is possible to verify in Figure 4 that a low ratio between the pixel size and the fiber radius may result in an area of fiber pixels lower/higher than the fiber circumference areas. In the next section, the quantitative effect of the adopted strategy with respect to the computation of the fiber volume fraction is presented. For this purpose, the fiber volume fraction of the cropped regions computed using the pixels counting is then compared to those computed using the circumference areas.

## 4. Applying Proposed Algorithm in the Analysis of Cross-Section Micrographs

To provide a bank of images to be quantitatively characterized using the spatial descriptor functions mentioned in Section 2, three cross-section micrographs, from different samples of the 3D-printed carbon fiber-reinforced composite material, were used. The samples were extracted from specimens previously manufactured for tensile and compression tests. After cutting and mounting the samples, they were prepared with grinding papers and polishing suspensions using an automated machine equipped with programmable burst dispensing. The images were acquired using an optical microscope Olympus model DSX-HRSU equipped with a 5× magnification lens. The samples were labeled as TE-0-1-1, TE-90-4-1, and TE-90-4-2. The adopted alphanumeric sequence presents information of fiber orientation, feedstock material number, 3D-printed plate number, and specimen number.

**Figure 5:**
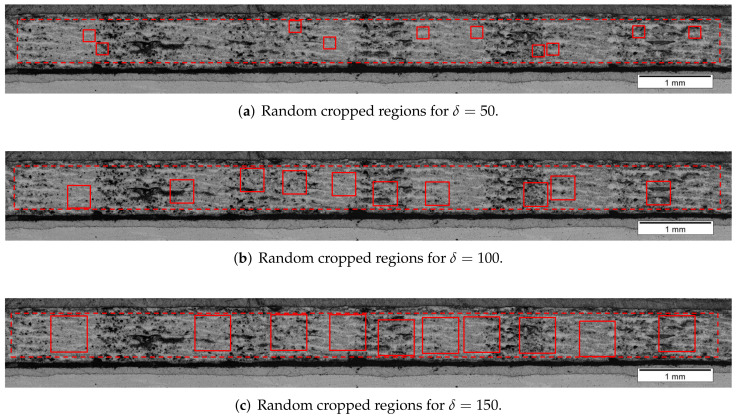
Cross-section micrograph of sample TE-90-4-1 with random cropped regions for δ=50, δ=100, and δ=150, and target fiber volume fraction $V_{f}^{*}=\left\{V_{f}:0.315\leq V_{f}\leq 0.320\right\}$.

The methodology summarized in Figure 2 was applied to sampling the regions from the main micrographs. To cover a wide range of sizes of cropped regions, i.e., from a minimum representative size up to the limit imposed by the vertical size of the cross-section micrograph, the parameter δ was set to δ={10,20,30,⋯,150}. For each parameter δ, a set of ten non-intersecting random regions was defined. Therefore, in the present work 150 regions were cropped from each one of the cross-section micrographs totalizing 450 analyzed images. Figure 5 shows the regions cropped from the cross-section micrograph of the sample TE-90-4-1 for δ=50, δ=100, and δ=150. The dashed red rectangles in Figure 5 show the area of search and the red squares depicts the random cropped regions. The target fiber volume fraction was set to $V_{f}^{*}=\left\{V_{f}:0.315\leq V_{f}\leq 0.320\right\}$, which is close to that experimentally measured and to that found in the literature.

**Figure 6:**
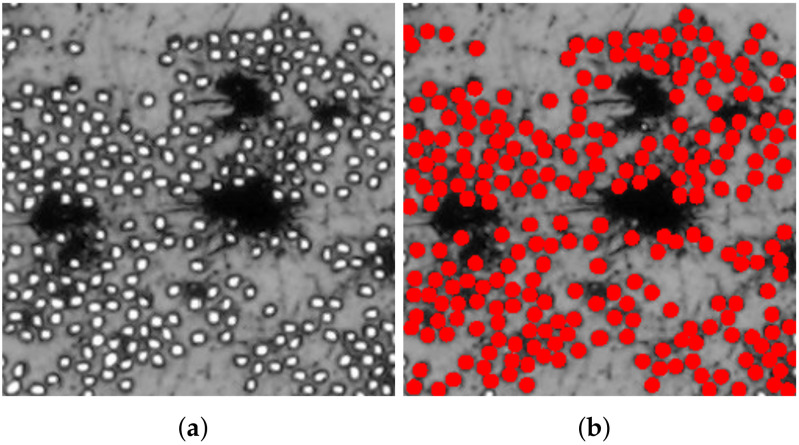
Application of the proposed algorithm in a cropped region of specimen TE-0-1-1: (**a**) reproduction of a cropped region from original micrograph used for computing the microstructure and (**b**) computed microstructure using the proposed algorithm. The computed fiber volume fraction is $V_{f}=31.85\%$.

**Figure 7:**
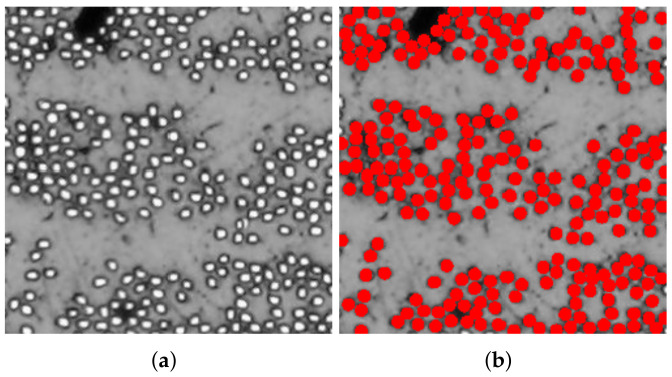
Application of the proposed algorithm in a cropped region of specimen TE-90-4-1: (**a**) reproduction of a cropped region from original micrograph used for computing the microstructure and (**b**) computed microstructure using the proposed algorithm. The computed fiber volume fraction is $V_{f}=31.96\%$.

**Figure 8:**
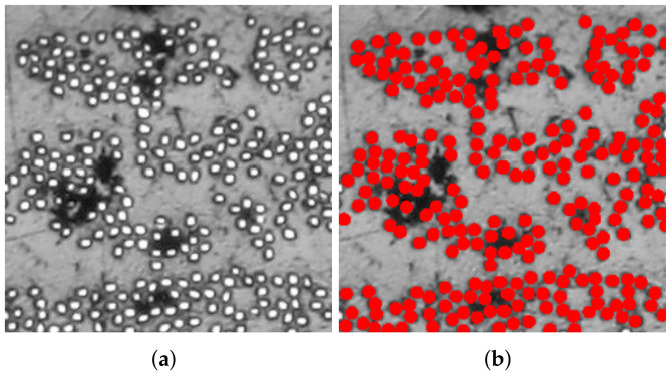
Application of the proposed algorithm in a cropped region of specimen TE-90-4-2: (**a**) reproduction of a cropped region from original micrograph used for computing the microstructure and (**b**) computed microstructure using the proposed algorithm. The computed fiber volume fraction is $V_{f}=31.71\%$.

Figure 6, Figure 7 and Figure 8 present reproductions of regions cropped from the cross-section micrographs samples TE-0-1-1, TE-90-4-1, and TE-90-4-2, in addition to their respective microstructures computed using the algorithm summarized in Figure 2. The size of the cropped regions in Figure 6, Figure 7 and Figure 8 is δ=50. To support the visualization of the computed microstructures, the fibers had their color changed to red in Figure 6b, Figure 7b and Figure 8b. The fiber volume fraction, calculated using the pixels counting method, is also shown for each one of the computed microstructures.

**Figure 9:**
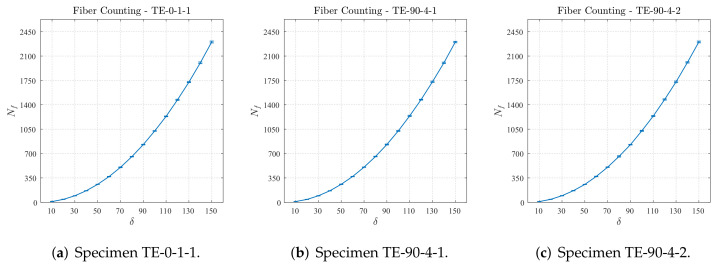
Number of fibers computed for different sizes of cropped regions.

Figure 9 presents the number of fibers that were identified in the cropped regions for each one of the analyzed cross-section micrographs. Since the range adopted for targeting the fiber volume fraction was considerably tight, the standard deviations, represented by the error bars, of the counted fibers for a given size δ are very small and can barely be seen in the curves for all samples. Another consequence of the tight targeted fiber volume fraction is the overall similarity between the curves obtained for all samples, i.e., the number of fibers identified in one of the samples, for a given size δ, is practically the same of those obtained for the other samples. From the methodology point of view, it can be said that the algorithm herein proposed to identify the fibers from cross-section micrographs is very robust and effective.

**Figure 10:**
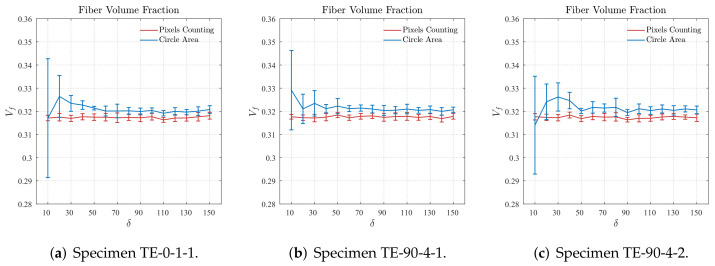
Fiber volume fraction computed for different sizes of cropped regions based on pixels counting and circumference areas.

Previously, in Section 3, the methodology applied for determining the fiber volume fraction was presented. In this case, the proportion of the number of pixels representing the fibers to the total number of pixels of cropped regions was computed. Therefore, prior to the statistical characterization of the cropped regions, the effect of the adopted strategy in comparison to the fiber volume fraction computed using circumference areas was verified. Figure 10 displays the mean and standard deviation (represented by the error bars) values for the fiber volume fraction, computed using both the pixels counting and circumference areas, obtained for the regions cropped from the cross-section micrographs samples TE-0-1-1, TE-90-4-1, and TE-90-4-2 for different δ sizes.

As verified in Figure 10, for small values of δ the standard deviation of the fiber volume fraction computed using the circumference areas is particularly high although the mean values are slightly close to those obtained from the pixels counting. For all the samples in Figure 10, it is verified that the fiber volume fraction obtained for the cropped regions with size values of δ≥50 present suitable results, i.e., the plots of fiber volume fraction computed using both methods are very close for size values of δ≥50. In addition to the points previously noted, the results shown in Figure 10 also illustrate the robustness and effectiveness of the herein proposed algorithm to identify fibers from cross-section micrographs. Analogously to that verified in Figure 9, observing the curves related to the pixels counting in Figure 10 it is possible to verify that the resulting fiber volume fraction is practically the same for all sizes δ of the cropped regions. Small values of standard deviation for a given δ is also observed.

## 5. Statistical Characterization of Cross-Section Micrographs

In Section 2, spatial descriptor functions used to quantitatively characterize distributions were introduced. The criteria used in both techniques for characterizing a distribution according to its periodicity and agglomeration were also presented. In the present section, the bank of images obtained from the cross-section micrographs samples TE-0-1-1, TE-90-4-1, and TE-90-4-2, for different sizes δ and with targeted fiber volume fractions, are quantitatively characterized according to the criteria adopted for the nearest neighbor distance and second-order intensity function. As previously noted, for every size δ, ten regions were cropped from a given main region of the cross-section micrograph. Therefore, the mean values of the respective functions computed for all cropped regions, for a given size δ, are plotted in the following figures. In the same curves, the error bars represent the standard deviation of their respective functions computed for the cropped regions given a size δ.

### 5.1. Nearest Neighbor Distance Characterization

**Figure 11:**
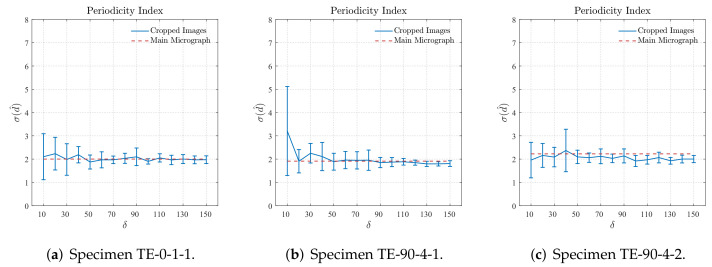
Standard deviation of nearest neighbor distance $\hat{d}$ computed for different sizes of cropped regions.

Figure 11 shows the results for the periodicity characterization obtained computing the standard deviation of nearest neighbor distance $\hat{\sigma }\left(\hat{d}\right)$ for different sizes δ of the cropped regions. A reference value of the standard deviation ${\hat{\sigma }}_{M}\left(\hat{d}\right)$ computed for the main region of the cross-section micrograph, i.e., the standard deviation ${\hat{\sigma }}_{M}\left(\hat{d}\right)$ computed for the whole area of search, exemplified in Figure 5, is also plotted in Figure 11. A preliminary inspection on the results shown in Figure 11 confirms that the fiber arrangement of the 3D-printed reinforced layers is non-periodic since ${\hat{\sigma }}_{M}\left(\hat{d}\right)\approx 1.9$. It is also verified in Figure 11a that the results obtained for cropped regions with size δ≥60 converge to those obtained for the main region of the cross-section micrograph from sample TE-0-1-1. From the results obtained for the samples TE-90-4-1, shown in Figure 11b, and TE-90-4-2, shown in Figure 11c, it is possible to verify that the cropped regions with size δ≥50 are able to represent their respective main regions of the cross-section micrograph according to the periodicity criteria adopted for the nearest neighbor distance.

**Figure 12:**
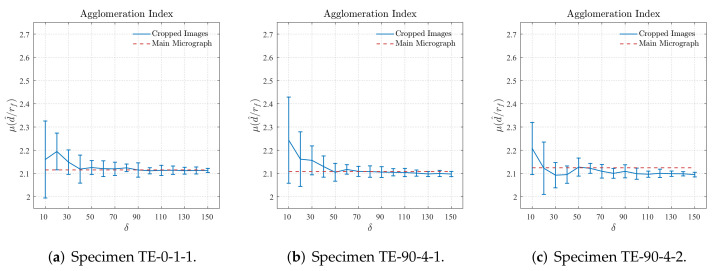
Mean value of ratio $\hat{d}/r_{f}$ computed for different sizes of cropped regions.

Figure 12 displays the results for the agglomeration characterization obtained computing the mean value of nearest neighbor distance $\hat{\mu }\left(\hat{d}/r_{f}\right)$ for different sizes δ of the cropped regions. Analogously to the results presented for the periodicity characterization, the mean value ${\hat{\mu }}_{M}\left(\hat{d}/r_{f}\right)$ computed for the main region of the cross-section micrograph is also plotted in Figure 12. The results obtained for the main region of the cross-section micrograph confirm that the fiber arrangement of the 3D-printed reinforced layers has a high degree of agglomeration since ${\hat{\mu }}_{M}\left(\hat{d}/r_{f}\right)\approx 2.1$. It is verified in Figure 12a,b that the results obtained for cropped regions with size δ≥50 converge to those obtained for the main regions of the cross-section micrographs from samples TE-0-1-1 and TE-90-4-1. In Figure 12c, it is possible to verify that the results obtained for the cropped regions converge to a value slightly lower than that obtained for the main region of the cross-section micrograph of sample TE-90-4-2. However, it is also possible to observe that the results obtained for the cropped regions with size δ=50 and δ=60 are very close to that obtained for the main region of the cross-section micrograph. Based on the previous notes, it is suggested that cropped regions with size δ=50 or δ=60 are suitable to represent their respective main regions of the cross-section micrograph according to the agglomeration criteria adopted for the nearest neighbor distance.

### 5.2. Second-Order Intensity Function Characterization

**Figure 13:**
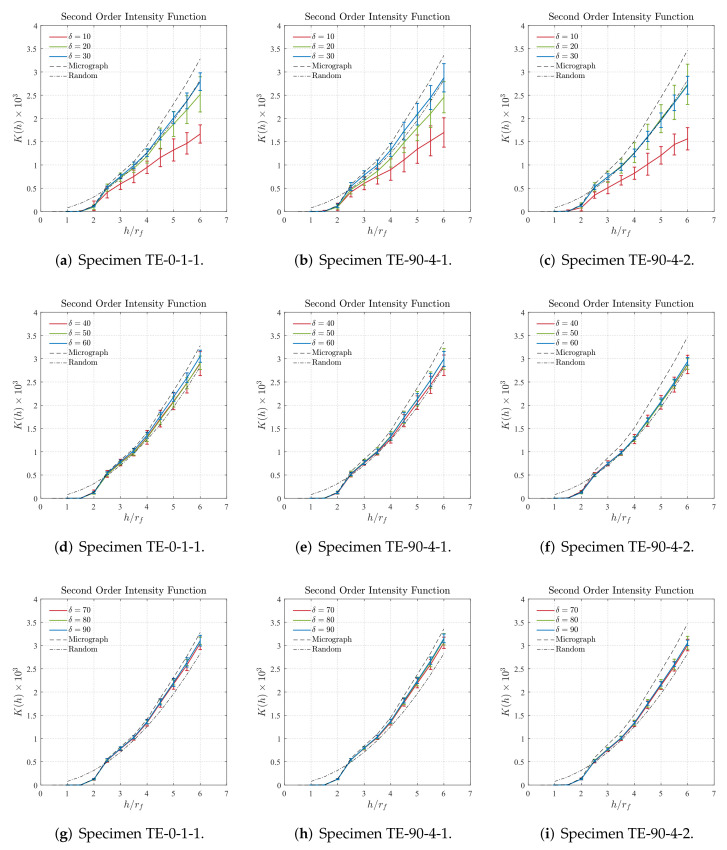
Second-order intensity function obtained for cropped regions of size δ=[10,90], as well as for the main region of the cross-section micrograph (dashed lines) and for a complete random distribution (dash-dotted lines).

**Figure 14:**
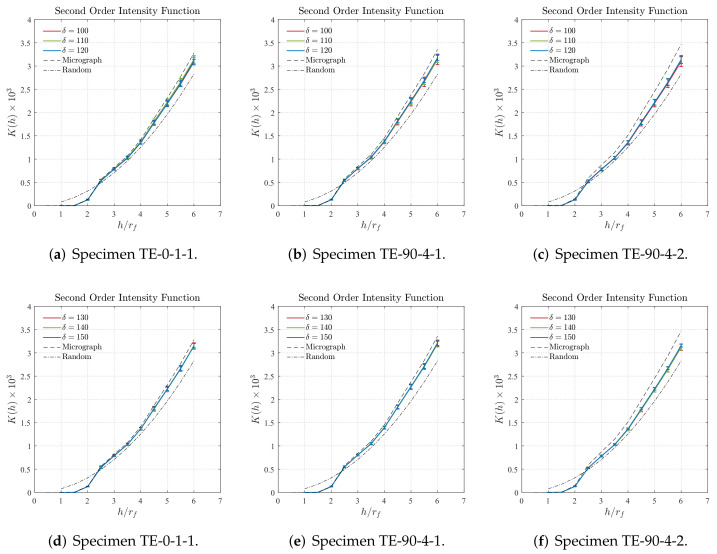
Second-order intensity function obtained for cropped regions of size δ=[100,150], as well as for the main region of the cross-section micrograph (dashed lines) and for a complete random distribution (dash-dotted lines).

Figure 13 and Figure 14 present the plots $K\left(h\right)\times h/r_{f}$ computed for the cropped regions of the cross-section micrographs, as well as those computed for the main region of the cross-section micrograph, represented by the dashed lines, and also for a complete random distribution, represented by the dash-dotted lines. To facilitate the visualization, and consequently the analysis of the curves, the results for different size of δ were plotted in groups of three in addition to the results plotted for the main region and those for the random distribution. From the overall results presented in Figure 13 and Figure 14, it can be first verified that the results obtained for all the cropped regions, in addition to those obtained for the main cross-section micrographs, have characteristics of non-periodic distributions since all the curves are monotonic positive, i.e., it is not possible to identify stair-shaped regions. Additionally, it is also verified that all the results obtained for the main region of the cross-section micrographs are above those obtained for random distributions which means that the fibers have some degree of agglomeration or clustering. Both characteristics were visually identified throughout the cross-section micrographs analysis performed prior to the statistical characterization.

### 5.3. Statistical Characterization Discussion

First, it is worth mentioning that the results presented in Section 5.1 and Section 5.2 show that the characteristics of the fiber distribution on the 3D-printed reinforced layers obtained using the Nearest Neighbor Distance are totally in accordance with those obtained with the Second-Order Intensity Function. In other words, it is a confirmation that both techniques can properly capture the main characteristics of the fiber distribution found on 3D-printed fiber-reinforced thermoplastics. According to the periodicity criteria adopted for the Nearest Neighbor Distance, cropped regions with size δ≥50 are representative of their respective main regions of the cross-section micrograph, which is verified in Figure 11. Additionally, according to the results obtained using the agglomeration criteria adopted for the same spatial descriptor, and shown in Figure 12, it is suggested that cropped regions with size δ=50 or δ=60 are suitable to represent their respective main regions of the cross-section micrograph. Regarding the characterization carried out adopting the Second-Order Intensity Function, the results are not entirely straightforward requiring a more developed discussion, which is given as follows.

From the results shown in Figure 13a–c it is suggested that cropped regions with small sizes δ are not recommended to represent the main region of the cross-section micrographs, according to the Second-Order Intensity Function. For instance, the results obtained for the cropped regions with δ=10 are considerably far from those obtained for the main cross-section micrograph mostly for higher values of the $h/r_{f}$. Similar behavior is identified for the results obtained for the cropped regions with size δ=20 although for the sample TE-90-4-2 the results are significantly better than those obtained for δ=10. For the cropped regions with size δ=30, the results are slightly better than those obtained for the cropped regions with size δ=10 and δ=20. However, it can be verified that they are closer to those obtained for random distributions than to those obtained for the main region for the cross-section micrographs.

The results shown in Figure 13d–f, indicate that cropped regions with sizes δ=50 and δ=60 can be suitable to represent the main region of their respective cross-section micrographs. This is verified more specifically in Figure 13d,e where the curves plotted for the sizes δ=50 and δ=60 are amid the curves obtained for the main region of cross-section micrograph and for the random distribution. The results obtained for the cropped regions with size δ=40, although can be also considered to be good representations, are closer to the results obtained for the random distributions than to those obtained for the main region of the cross-section micrographs. Similar behavior is identified for the results obtained for the sample TE-90-4-2 and shown in Figure 13f where it can be verified that all plots are closer to the results obtained for the random distributions than to those obtained for the main region of the cross-section micrographs.

The results shown in Figure 13g–i reveal some degree of convergence between the plotted curves since they are very close to each other. More specifically, the results obtained for the samples TE-0-1-1, displayed in Figure 13g, and TE-90-4-1, displayed in Figure 13h, are particularly close to those obtained for the main region of the cross-section micrographs. Similar behavior is seen for the results obtained for the cropped regions with sizes δ=100, δ=110, and δ=120 and displayed in Figure 14a–c. In this case, the difference between the curves obtained for the cropped regions barely can be seen. Additionally, it must be noted that the results in Figure 14a,b are substantially close to those obtained for the respective main region of the cross-section micrograph. Regarding the results displayed in Figure 14 for the cropped regions with sizes δ=130, δ=140, and δ=150, the obtained curves can be considered well converged and considerably close to the those obtained for the main region of the cross-section micrograph.

Based on the discussion presented above, it is possible to conclude that cropped regions with size δ=50 are statistically equivalent to their respective main regions of the cross-section micrographs. In other words, it means that cropped regions with a minimum size δ=50, from any one of the three characterized specimens, represent the microstructure of the 3D-printed fiber-reinforced thermoplastics herein analyzed. To summarize this point, Table 3 describes the minimum recommended size δ of the cropped regions, which are statistically representative of the microstructures found in the analyzed specimens. In addition, attempting to verify the validity of the proposed methodology in terms of microstructure representation, the results summarized in Table 3 were compared to those obtained by Trias et al. in [51]. Although in their work a carbon fiber-reinforced epoxy was investigated, the results herein obtained can be considered in agreement with theirs, where a minimum recommended size for a representative volume was found to be δ=50.

## 6. Homogenized Properties

According to the literature, different techniques were adopted to verify the accuracy of volumes that are equivalent to the whole non-periodic domain. Typically, investigations about the effect of the geometric characteristics of a representative volume on the resulting homogenized elastic properties were carried out [28,29,39,40,43,46,47,48,51,52,55,56,62]. From this point of view, it can be realized that the statistically equivalent volumes herein determined shall also be representative in terms of homogenized elastic properties. Therefore, attempting to demonstrate the effectiveness of the proposed methodology, the homogenized elastic properties of 3D-printed continuous carbon fiber-reinforced thermoplastics are determined and compared to those experimentally measured, according to the data available in the literature. For the numerical computations, the Asymptotic Homogenization method is employed. Detailed information about the adopted representative equivalent volumes, as well as about the finite element models, are given as follows.

### 6.1. Numerical Modeling

As verified in Section 5, equivalent volumes with size δ≥50 can represent the microstructure of 3D-printed fiber-reinforced thermoplastics in terms of fiber distribution. Thus, to use computationally feasible models on the analysis, and be representative in terms of variability, representative equivalent volumes with size δ=[10,60] obtained for the sample TE-90-4-1 are used. More specifically, the cropped regions whose results were the closest (with respect to the chosen statistical criteria) to the result obtained for the main region of the respective cross-section micrograph were selected. Table 4 describes in detail the information about the selected cropped regions.

The implementation of the Asymptotic Homogenization technique followed the methodology fully described in [63], which was also applied in [26]. More specifically, the models were implemented in ABAQUS^®^ software with the application of periodic boundary conditions, assuming that the fiber distribution determined in the previous section are equivalent to those found in the cross-section micrographs. It is worth noting that the application of periodic boundary conditions on ABAQUS^®^ are not directly available. In this case, it requires the definition of linear constraints using equations to determine the relative motion between the degrees of freedom of two or more nodes, as can be seen in [63]. In addition, the fibers were assumed as continuous in the finite element discretization, which leads the solution to be independent of the coordinate along the fiber direction. Therefore, it was not necessary to refine the mesh along this direction. However, it is worth remarking that in other different cases, e.g., modeling short fiber-reinforced composite materials and/or composite materials with inclusions, the mesh refinement is important in all directions. Table 5 presents the mechanical properties of the carbon fiber and thermoplastic matrix [7,8] adopted in the finite element discretization.

**Figure 15:**
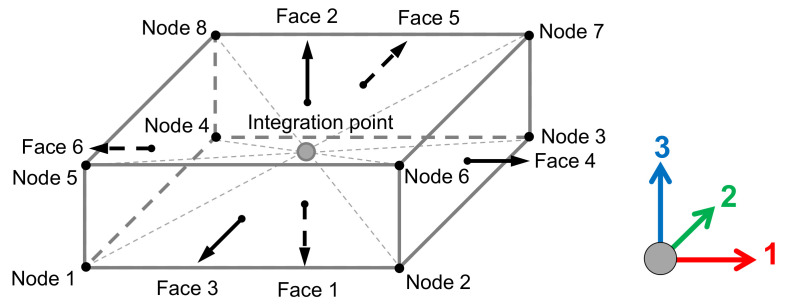
Eight-node linear hexahedral elements with reduced integration of type C3D8R available on ABAQUS^®^.

The finite element model uses eight-node linear hexahedral elements with reduced integration of type C3D8R available on ABAQUS^®^. More details about the element formulation is displayed in Figure 15 and additional information can be found in [64]. Table 6 summarizes details of the finite element discretization used in the Asymptotic Homogenization models.

**Figure 16:**
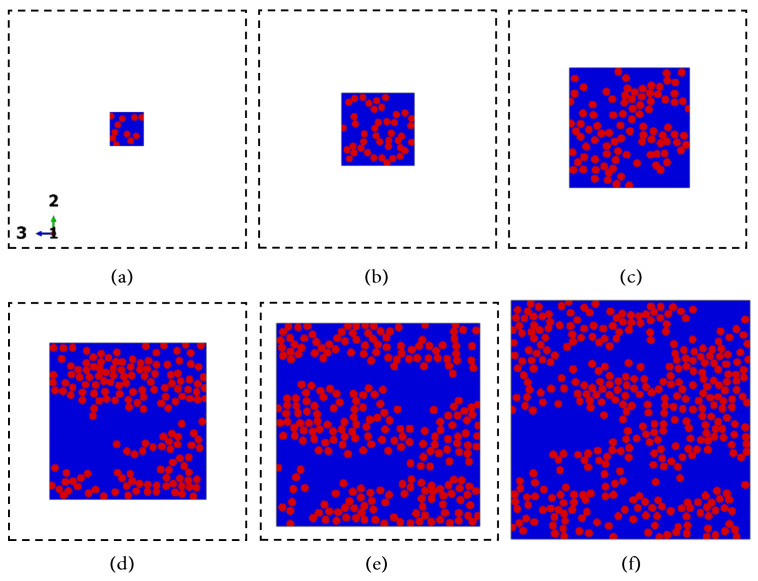
Discretized cropped regions with size δ=10 (**a**), δ=20 (**b**), δ=30 (**c**), δ=40 (**d**), δ=50 (**e**) and δ=60 (**f**). For all the finite element models, the element size corresponds to one pixel size according to their respective computed microstructures.

As an illustration of the finite element models adopted in the current analysis, Figure 16 displays the finite element discretization applied to the cropped regions with size δ=[10,60], where the thermoplastic matrix is represented in blue, and the fibers are represented in red. In Figure 16 the cropped regions were discretized according to the details in Table 6. To provide a direct comparison between the size of the discretized regions, dashed line squares with size δ=60 were also included in Figure 16. For all the finite element models herein adopted, the element size corresponds to one pixel size according to their respective computed microstructures.

### 6.2. Numerical Results and Discussion

Table 7 summarizes the homogenized mechanical properties obtained for the 3D-printed continuous carbon fiber-reinforced thermoplastics using the Asymptotic Homogenization technique applied to the cropped regions listed in Table 4. As already expected, the variations on the homogenized longitudinal elastic modulus $E_{1}$ are barely noticeable. In contrast to the transverse in-plane elastic modulus $E_{2}$, which seemed to be not affected by the size of the equivalent volume, the transverse out-of-plane elastic modulus $E_{3}$ presented some variation for the equivalent volume with size δ=40 that is not observed for those with size δ=50 and δ=60. Nevertheless, it is worth remarking that the variations observed for the homogenized extension moduli in function of the size of the equivalent volume are considerably low being less than 3.5% for all the moduli presented in Table 7.

In addition, it can be seen from Table 7 that the results obtained for the transverse out-of-plane shear elastic modulus $G_{23}$, are not highly affected by the equivalent volume size where the maximum variation found is less than 3%. For their part, the in-plane shear elastic modulus $G_{12}$ and the transverse out-of-plane shear elastic modulus $G_{13}$ are slightly more affected by the equivalent volume size. However, the maximum variation computed for the homogenized shear elastic moduli was 7.5% for $G_{12}$ and 10.4% for $G_{13}$ which are not considerably high since they are at the same level of variations occasionally found on experimental testing data, when comparing the results from one sample to another. Furthermore, these maximum values of variation are observed for the equivalent volume with size δ=40 in comparison to that with size δ=10 for both $G_{12}$ and $G_{13}$. Comparing the results obtained for the equivalent volume with size δ=40, with the results obtained for the equivalent volumes with sizes δ=50 and δ=60, these variations are strongly reduced.

Analogously, the results obtained for the Poisson ratios seemed to be not highly affected by the size of the equivalent volume. Some variations are observed, mostly for the homogenized Poisson ratios $\nu_{13}$ and $\nu_{23}$, though the maximum difference was found to be 3.1%, 5.9%, and 4.9% respectively for $\nu_{12}$, $\nu_{13}$, and $\nu_{23}$. These values can be normally acceptable since they were found for the representative equivalent volume with size δ=40 in comparison to the representative equivalent volume with size δ=10. As with the results presented for the homogenized shear moduli, comparing the results obtained for the representative equivalent volume size δ=40 with those obtained for the volumes with δ=50 and δ=60 these variations are strongly reduced. Therefore, it can be concluded from Table 7 that the values obtained for different sizes slightly differ in value from one to another which results in a coefficient of variation (CoV) that is particularly small. As expected, it is also verified that the results obtained for the representative equivalent volume with size δ=50 are considerably close to those obtained for the representative equivalent volume with size δ=60 indicating that some degree of convergence has been achieved.

To demonstrate the validity of the proposed methodology, the homogenized elastic properties $E_{1}$, $E_{2}$, and $G_{12}$ obtained for the representative equivalent volumes with size δ=50 and δ=60 were compared in detail with those experimentally measured from specimens tested in tension (longitudinal and transverse) and in-plane shear as presented in [17]. To offer more understanding of the validity of the proposed methodology, the results herein obtained were also compared to those numerically determined in [17] using a perfect square unit cell representing the fiber arrangement. Table 8 summarizes the comparison.

It can be verified from Table 8 that the differences between the homogenized properties and experimental data computed for $E_{1}$ and $G_{12}$ are negligible for both representative equivalent volumes. The differences computed for $E_{2}$ are not negligible although they are relatively small, especially for the representative equivalent volume with size δ=50. From the overall results point of view, it can be said that the homogenized elastic properties $E_{1}$, $E_{2}$, and $G_{12}$ accurately agreed with their respective elastic properties experimentally measured. The same agreement is verified when comparing the results obtained for the representative equivalent volumes with those obtained using the perfect square unit cell, which corroborates to the validity of the methodology herein proposed. Nevertheless, at this point a relevant discussion about the fiber distribution is worth of mentioning. According to the literature, Pyrz [56] evaluated the influence of the constituent spatial distribution on the field quantities and concluded that the variability of stresses, in particular the maximal radial stresses, was considerably affected as the constituent spatial distribution changed. In similar studies, Matsuda et al. [62] and Trias et al. [51] showed that the “randomness” of a fiber distribution strongly affects the microscopic distribution of stresses and strains. Therefore, even though the results obtained for the representative equivalent volumes are particularly close to those obtained using the perfect square unit cell, the proposed methodology provides equivalent volumes considerably more representative in terms of both fiber distribution and stresses/strains distribution at microscopic level.

## 7. Conclusions

In the present work, a methodology based on the statistical characterization of the microstructure found in 3D-printed fiber-reinforced thermoplastics was proposed. Since the microstructure of these materials cannot be considered completely random regardless of other factors, e.g., the manufacturing process, which can affect the fiber spatial and pores distribution, a different strategy was adopted and a methodology to define a region that could represent the main cross-section micrograph, in terms of size and fiber arrangement, was defined. For this purpose, spatial descriptor functions, namely the nearest neighbor distance and the second-order intensity function, were adopted to statistically characterize the cropped regions. Then, a simple algorithm for processing the cross-section micrographs, i.e., for defining the regions to be cropped, identifying the fibers and creating a bank of images, was presented. In view of the relatively low fiber volume fraction found in 3D-printed fiber-reinforced thermoplastics, the proposed methodology was highly suitable to define the microstructure of sub-volumes accounting for their specific characteristics, such as some degree of non-periodicity and agglomeration.

In the statistical characterization, a parametric study was conducted using the cross-section micrographs from three different samples. In this context, several images of sub-regions, previously cropped from the original cross-section micrographs, were used to determine both the fiber arrangement and the minimum size of the sub-region which are statistically equivalent to the whole material. From the obtained results, it was confirmed that the fiber arrangement was neither periodic nor completely random. In addition, it is also concluded that representative equivalent volumes with size δ=50 can reproduce with strong fidelity the fiber spatial distribution of the 3D-printed fiber-reinforced thermoplastics. To verify the effectiveness of the proposed methodology in terms of microstructure representation, the obtained results were compared to those available in the literature for carbon fiber-reinforced epoxy, since there is still a lack of these studies for 3D-printed fiber-reinforced thermoplastics. In any case, the results herein obtained were considered in agreement with theirs in terms of a minimum equivalent volume size required to represent the spatial fiber distribution of fiber-reinforced materials.

In complement to the validation in terms of fiber distribution, the effectiveness of the proposed methodology was also validated in terms of homogenized elastic properties, which were computed using different sizes of sub-domains. The obtained results were then compared to those available in the literature showing excellent agreement in view of the computed variations. In this context, the smaller adopted cropped region that represents the main micrograph better from the computational micromechanics point of view, since less computational efforts are required. However, for particular small sizes of sub-domains, some variations were found when comparing the homogenized properties with those computed from experimental results. More specifically, it was concluded that small representative equivalent volumes may present some variations in the homogenized elastic properties of 3D-printed fiber-reinforced thermoplastics. Although these variations were relatively small, the stress/strain distribution at the microscopic level can be highly affected by the fiber arrangement as seen in the literature survey.

As mentioned above, specific characteristics found in the microstructures of 3D-printed fiber-reinforced thermoplastics, such as degrees of non-periodicity and agglomeration as well as the fiber distribution, are of great importance, mostly when conducting mechanism-based failure analyses, which have the stresses/strains at microscopic level as the main input. In this line of thought, it is worth noting that voids should be also contemplated in this analysis, since their content can be particularly high, which is aggravated by their irregular distribution. Therefore, an extension of the methodology herein presented to include more than two constituents is part of future research. In terms of the proposed algorithm, its extension is particularly straightforward due to its high flexibility. Nevertheless, alternative imaging techniques may be required to provide clearer cross-section micrographs, consequently improving the efficiency of the proposed methodology.

## Figures and Tables

**Table 1:** Adopted criteria for quantitatively characterizing a fiber distribution using the nearest neighbor distances.

Characteristic	Criteria
Agglomerated	$\hat{\mu }\left(\hat{d}/r_{f}\right)\to 2$
Dispersed	$\hat{\mu }\left(\hat{d}/r_{f}\right)>2$
Periodic	$\hat{\sigma }\left(\hat{d}\right)\to 0$
Non-periodic	$\hat{\sigma }\left(\hat{d}\right)>0$

**Table 2:** Conditions for characterizing a fiber arrangement as agglomerated/dispersed and periodic/non-periodic based on the second-order intensity function.

Characteristic	Function K(h)
Agglomerated	$K\left(h\right)>K_{p}$
Dispersed	$K\left(h\right)<K_{p}$
Periodic	Stair-shaped
Non-periodic	Monotonic Positive

**Table 3:** Summary of minimum recommended size δ according to the adopted criteria.

Specimen	Nearest Neighbor Distance		2nd Order Intensity Function	
	Periodicity	Agglomeration	Periodicity	Agglomeration
TE-0-1-1	δ≥60	δ≥40	δ≥10	δ≥50
TE-90-4-1	δ≥50	δ≥50	δ≥10	δ≥50
TE-90-4-2	δ≥50	δ≥50	δ≥10	δ≥40

**Table 4:** Cropped regions and their respective spatial descriptors.

Size δ	Cropped Region	$V_{f}$	$\hat{\sigma }\left(\hat{d}\right)$	$\hat{\mu }\left(\hat{d}/r_{f}\right)$	K(h)h=6
10	CR05	31.72%	2.0065	2.2921	1222
20	CR05	31.66%	1.8620	2.0823	2145
30	CR07	31.81%	1.8499	2.1752	2668
40	CR07	31.51%	1.8445	2.0874	3072
50	CR01	31.96%	2.0606	2.1472	3035
60	CR02	31.75%	1.8470	2.1051	2926

**Table 5:** Adopted mechanical properties of carbon fiber and resin matrix [7,8].

Mechanical Properties	Carbon Fiber	Thermoplastic Matrix
Longitudinal Modulus [GPa]	230	3.2
Transverse Modulus [GPa]	15	3.2
Longitudinal Shear Modulus [GPa]	15	1.2
Transverse Shear Modulus [GPa]	15	1.2
Poisson ratio	0.2	0.3

**Table 6:** Finite element discretization details.

Size δ	Number of Elements	Number of Nodes
10	2601	5408
20	10,201	20,808
30	22,801	46,208
40	40,401	81,608
50	63,001	127,008
60	90,601	182,408

**Table 7:** Results obtained for the homogenized mechanical properties.

Size δ	$E_{1}$ [MPa]	$E_{2}$ [MPa]	$E_{3}$ [MPa]	$G_{12}$ [MPa]	$G_{13}$ [MPa]	$G_{23}$ [MPa]	$\nu_{12}$	$\nu_{13}$	$\nu_{23}$
10	75,187	5189	5153	2399	2292	2024	0.267	0.185	0.339
20	75,067	5165	5194	2361	2390	2032	0.269	0.182	0.336
30	75,397	5147	5178	2314	2360	2048	0.269	0.182	0.338
40	74,718	5156	5333	2231	2531	1991	0.276	0.175	0.323
50	75,748	5209	5262	2340	2454	2026	0.271	0.179	0.330
60	74,945	5172	5188	2364	2387	2036	0.269	0.183	0.336
**Avg**	75,177	5173	5218	2335	2402	2026	0.270	0.181	0.334
**CoV**	0.48%	0.44%	1.28%	2.49%	3.40%	0.95%	1.1%	2.00%	1.85%

**Table 8:** Comparison of homogenized elastic properties with experimental data where $\Delta =100\times \left|\left(\mathrm{EXP}\;-\;\mathrm{HM}\right)/\mathrm{EXP}\right|$ with EXP the experimental data and HM the homogenized properties computed for the respective representative equivalent volume size.

Mech. Prop.	Experimental Data	Perfect Square Unit Cell		Representative Equivalent Volume			
	From [17]	From [17]	Δ [%]	δ=50	Δ [%]	δ=60	Δ [%]
$E_{1}$ [MPa]	74,970	76,401	1.91%	75,748	1.04%	74,945	0.03%
$E_{2}$ [MPa]	5529	5257	4.92%	5209	5.79%	5172	6.46%
$G_{12}$ [MPa]	2352	2153	8.46%	2340	0.51%	2364	0.51%

## Data Availability

The MATLAB code is available via a public Github repository at https://github.com/tadutra/periodic-equiv-volumes, accessed on 1 February 2022, which may be updated with bug fixes and new features based on state of the art of image processing. Should users wish to discuss the program or report bugs, they can contact the corresponding author by e-mail at: thiagoassis.dutra@gmail.com.
